# Metabolomic Profiling of Portal Blood and Bile Reveals Metabolic Signatures of Primary Sclerosing Cholangitis

**DOI:** 10.3390/ijms19103188

**Published:** 2018-10-16

**Authors:** Pamela S. Tietz-Bogert, Minsuk Kim, Angela Cheung, James H. Tabibian, Julie K. Heimbach, Charles B. Rosen, Madhumitha Nandakumar, Konstantinos N. Lazaridis, Nicholas F. LaRusso, Jaeyun Sung, Steven P. O’Hara

**Affiliations:** 1 Division of Gastroenterology and Hepatology, Mayo Clinic College of Medicine and Science, Rochester, MN 55905, USA; tietz.pamela@mayo.edu (P.S.T.-B.); Cheung.Angela@mayo.edu (A.C.); JTabibian@dhs.lacounty.gov (J.H.T.); lazaridis.konstantinos@mayo.edu (K.N.L.); larusso.nicholas@mayo.edu (N.F.L.); 2 Center for Cell Signaling in Gastroenterology, Mayo Clinic, Rochester, MN 55905, USA; 3 Microbiome Program, Center for Individualized Medicine, Mayo Clinic, Rochester, MN 55905, USA; Kim.Minsuk@mayo.edu; 4 Division of Surgical Research, Department of Surgery, Mayo Clinic, Rochester, MN 55905, USA; 5 Division of Gastroenterology, Department of Medicine, Olive View-UCLA Medical Center, Sylmar, CA 91342, USA; 6 Division of Transplant Surgery, Mayo Clinic College of Medicine, Rochester, MN 55905, USA; Heimbach.Julie@mayo.edu (J.K.H.); Rosen.Charles@mayo.edu (C.B.R.); 7 Seres Therapeutics, Cambridge, MA 02139, USA; mnandakumar@serestherapeutics.com

**Keywords:** primary sclerosing cholangitis (PSC), portal venous blood, bile, metabolomics, enterohepatic circulation

## Abstract

Primary sclerosing cholangitis (PSC) is a pathogenically complex, chronic, fibroinflammatory disorder of the bile ducts without known etiology or effective pharmacotherapy. Emerging in vitro and in vivo evidence support fundamental pathophysiologic mechanisms in PSC centered on enterohepatic circulation. To date, no studies have specifically interrogated the chemical footprint of enterohepatic circulation in PSC. Herein, we evaluated the metabolome and lipidome of portal venous blood and bile obtained at the time of liver transplantation in patients with PSC (*n* = 7) as compared to individuals with noncholestatic, end-stage liver disease (viral, metabolic, etc. (disease control, DC, *n* = 19)) and to nondisease controls (NC, living donors, *n* = 12). Global metabolomic and lipidomic profiling was performed on serum derived from portal venous blood (portal serum) and bile using ultraperformance liquid chromatography-tandem mass spectrometry (UPLC-MS/MS) and differential mobility spectroscopy-mass spectroscopy (DMS-MS; complex lipid platform). The Mann–Whitney *U* test was used to identify metabolites that significantly differed between groups. Principal-component analysis (PCA) showed significant separation of both PSC and DC from NC for both portal serum and bile. Metabolite set enrichment analysis of portal serum and bile demonstrated that the liver-disease cohorts (PSC and DC) exhibited similar enrichment in several metabolite categories compared to NC. Interestingly, the bile in PSC was uniquely enriched for dipeptide and polyamine metabolites. Finally, analysis of patient-matched portal serum and biliary metabolome revealed that these biological fluids were more homogeneous in PSC than in DC or NC, suggesting aberrant bile formation and enterohepatic circulation. In summary, PSC and DC patients exhibited alterations in several metabolites in portal serum and bile, while PSC patients exhibited a unique bile metabolome. These specific alterations in PSC are amenable to hypothesis testing and, potentially, therapeutic pharmacologic manipulation.

## 1. Introduction

Primary sclerosing cholangitis (PSC) is a chronic, progressive liver disease characterized by inflammation and fibrosis of the intra- and/or extrahepatic bile ducts. This cholestatic disease may progress to cirrhosis and liver failure, and patients are at increased risk for the development of malignancies, particularly cholangiocarcinoma [1,2]. Currently, the pathogenic mechanisms of PSC are poorly understood. The only therapy that improves the survival of PSC patients is liver transplant; thus, despite being a rare disease, PSC is the fifth most common indication for liver transplant in the United States [3]. Improving our understanding of PSC pathogenesis is necessary for developing effective and targeted medical therapies.

The enterohepatic circulation refers to molecule cycling between the liver and the intestine. In this bidirectional relationship, numerous endo-/exogenous metabolites and bioactive compounds are delivered to the gut via bile (e.g., bile acids and phospholipids), and, conversely, metabolites from the intestinal environment are continuously routed back to the liver via portal circulation [4]. Indeed, the liver receives 75%–80% of its blood supply from the portal vein, which collects blood from the small and large intestine, spleen, stomach, and pancreas [5,6]; the remaining 20%–25% is delivered via the hepatic artery. Upon arrival to the liver, arterial and portal blood mix in hepatic sinusoids where gut-derived molecules first encounter resident (sinusoidal endothelial, Kupffer, and stellate cells) and recruited (i.e., innate and adaptive immune) cells. The molecular constituents of blood freely pass through the fenestrated endothelial cells and interact with the basolateral domain of hepatocytes, where they may be absorbed, metabolized, and excreted into bile ducts via the hepatocyte canalicular (apical) membrane.

The gut–liver signaling axis has been proposed as a contributing factor for the initiation and/or progression of PSC. Although PSC is generally regarded as an idiopathic disease, several lines of evidence have suggested that the pathogenesis of PSC entails, in part, enterically derived microbial and nonmicrobial products, and downstream-signaling cascades. For example, the preponderance of inflammatory bowel disease (IBD) in patients with PSC [7,8] suggests a pathological link between gut and liver. More recently, it was demonstrated that the gut microbiota of PSC patients is distinct from those of patients with isolated IBD and healthy controls [9,10,11,12,13]. Moreover, studies of oral antibacterial therapy, including a recent randomized clinical trial using vancomycin, have demonstrated significant improvements in multiple disease markers in PSC patients [14,15]. However, the role of the enteric microbiota is complex; for example, not only can enteric microbial (and nonmicrobial) metabolites induce profibroinflammatory-signaling cascades in cultured biliary epithelial cells (i.e., cholangiocytes) [16,17] and rodent model systems [18], but the absence of microbiota in experimental germfree PSC models (i.e., Abcb4^−/−^ mouse) leads to more severe disease [19]. These studies support that intestinal health and microbial composition may play a role in PSC pathogenesis by altering the profile of microbially derived portal metabolites. While this gut–liver axis has been proposed as a major contributing factor in PSC etiopathogenesis, the hypothesis has not been to date directly addressed in patients.

The goal of this study was to identify a molecular profile within the enterohepatic circulation unique to PSC. To this end, we used global metabolomic and lipidomic approaches on serum derived from portal blood (portal serum) and bile collected at the time of transplant from: (i) seven patients with end-stage PSC, (ii) 19 patients with end-stage, non-PSC liver disease (viral, metabolic, etc. (disease control, DC, *n* = 19)), and (iii) 12 control, living donor subjects without liver disease (nondisease control, NC). While previous studies examined the metabolome in the peripheral serum of primary biliary cholangitis (PBC) and PSC patients [20,21], to our knowledge, this work is the first to explore the metabolomic profile of both portal serum and bile simultaneously, obtained at the time of living donor transplant, thereby allowing for more direct insight into enterohepatically circulated molecules. This study provides the first direct evidence that portal serum and bile metabolites from patients with PSC are different than those from patients with non-PSC cirrhosis, as well as from controls without liver disease. Furthermore, this study reveals metabolic pathways that are possibly disrupted uniquely in PSC patients, yielding potential targets for the development of novel therapies.

## 2. Results

### 2.1. Patient Characteristics

A total of 38 subjects were included in this metabolomic profiling study. Table 1 summarizes the clinical characteristics and liver biochemistries of the study participants. Consistent with established gender predispositions and association with IBD, the PSC group was predominantly male (4/7, ~57%) and 4/7 (~57%) had concurrent IBD. Additional characteristics such as age, ethnicity, and body mass index were generally similar across PSC, DC, and NC. Statistical comparisons of serum liver biochemistries revealed significantly higher alkaline phosphatase and total bilirubin levels in PSC and DC compared to NC (*p* < 0.05). While all liver-disease patients met criteria for liver transplant, the mean Model for End-Stage Liver Disease (MELD) scores were lower in the PSC patient population (*p* < 0.05) compared to DC. None of the subjects had previously undergone colectomy. Importantly, at the time of liver transplantation, some PSC and DC patients were receiving ursodeoxycholic acid (UDCA) therapy (2/7 (~28%) and 2/19 (~10%), respectively), and/or antibiotics (1/7 (~14%) and 2/19 (~21%), respectively).

### 2.2. Global Metabolomic Profiling to Identify Phenotype-Specific Chemical Species and Enriched Categories

Five liquid chromatography-mass spectrometry (LC-MS) runs (platforms) were utilized for each portal serum and bile sample: four ultraperformance liquid chromatography-tandem mass spectrometry (UPLC-MS/MS) runs for metabolomics (LC-MS-negative, -polar, -positive early, and -positive late) and one differential mobility spectroscopy (DMS)-MS run for complex lipids (lipidomics). A total of 1990 features were detected in portal serum: 671 features with known identity, 420 features with unknown identity, and the remaining 899 being complex lipids. A total of 2014 features were detected in bile: 690 features with known identity, 284 features with unknown identity, and the remaining 1040 being complex lipids.

**Figure 1:**
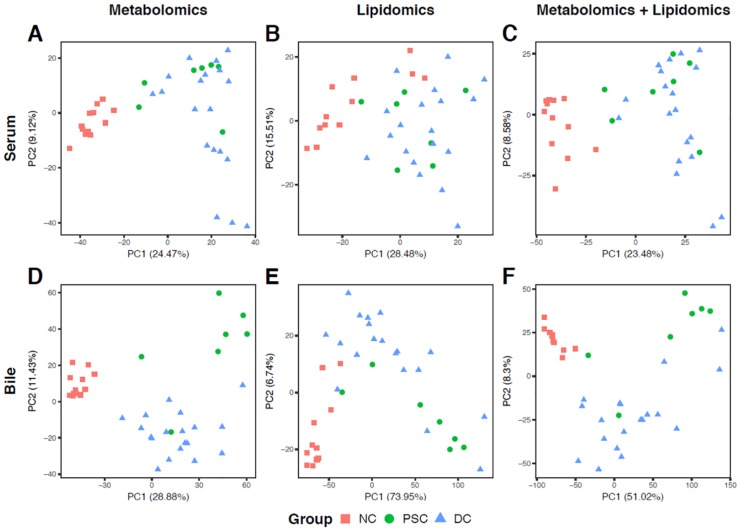
Principal-component analysis (PCA) ordination plots of metabolomic (**A**,**D**), lipidomic (**B**,**E**), and combined (**C**,**F**) profiles of portal serum (**A**–**C**) and bile (**D**–**F**) from patients with primary sclerosing cholangitis (PSC), cirrhotic liver disease (disease control, DC), and from heathy controls (nondisease control, NC). For the ordination of metabolomic data, relative intensities of both annotated and unannotated liquid chromatography-mass spectrometry (LC-MS) peaks were used.

Our principal-component analysis (PCA) plot of portal serum metabolomics revealed a clear separation between NC and liver-disease samples (PSC or DC) along PC 1 (Figure 1A) but less separation between the same sample groups according to serum lipidomics (Figure 1B) or when both platforms were combined (Figure 1C). PCA for bile metabolomics revealed clear separation between PC 1 and PC 2 for all three groups, with PSC and DC samples separating along PC 2 (only one PSC bile sample fell into the DC cluster; Figure 1D). PCA for bile lipidomics displayed noticeable separation between NC and liver disease samples, despite there being high intragroup sample variability, especially within the liver diseases (Figure 1E). As with the serum data, ordination of the combined bile metabolomic and lipidomic profiles showed less separation (Figure 1F).

**Figure 2:**
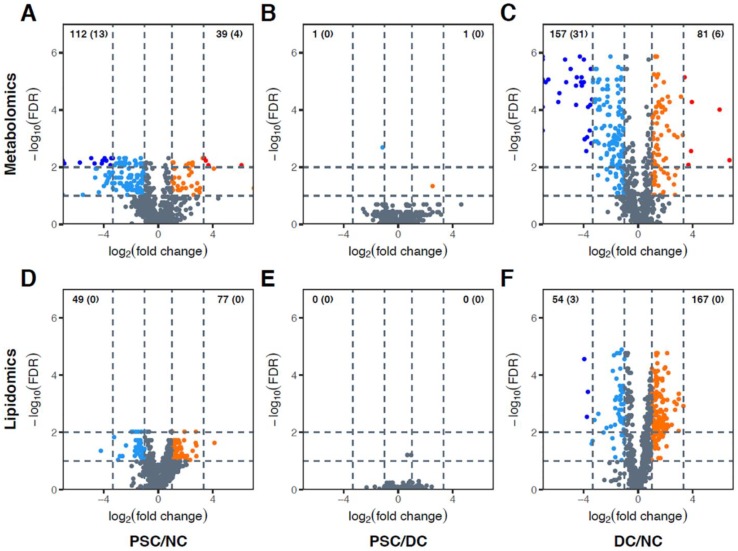
Volcano plots showing differentially abundant metabolites (**A**–**C**) and complex lipids (**D**–**F**) in portal serum. Every possible pair of groups, i.e., PSC versus NC (**A**,**D**), PSC versus DC (**B**,**E**), and DC versus NC (**C**,**F**), were compared. The horizontal axis represents direction and magnitude of change in relative intensities, and the vertical axis represents statistical significance of the change. Two different thresholds for fold change (FC) and false discovery rate (FDR) were used: (i) up- (orange) and downregulated (sky blue) metabolites (FC > 2 and FDR < 0.1); and (ii) very significantly up- (red) and downregulated (blue) metabolites (FC > 10 and FDR < 0.01). Note that very significantly up- and downregulated metabolites are subsets of up- and downregulated metabolites, respectively. Numbers shown in the upper-left and -right corners of each panel represent counts of up- and downregulated metabolites, respectively. Numbers in parentheses represent counts of very significantly up- or downregulated metabolites.

**Figure 3:**
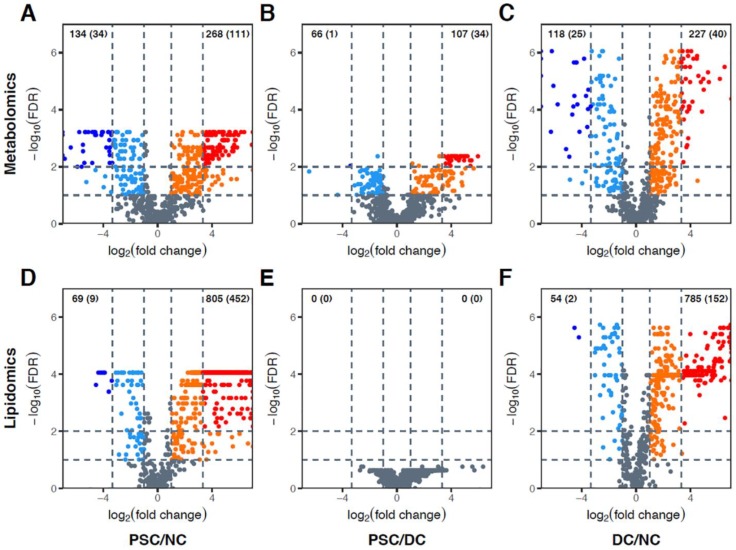
Volcano plots showing differentially abundant metabolites (**A**–**C**) and complex lipids (**D**–**F**) in bile. Every possible pair of groups, i.e., PSC versus NC (**A**,**D**), PSC versus DC (**B**,**E**), and DC versus NC (**C**,**F**), were compared. The horizontal axis represents the direction and magnitude of change in relative intensities, and the vertical axis represents statistical significance of the change. Two different thresholds for FC and FDR were used: (i) up- (orange) and downregulated (sky blue) metabolites (FC > 2 and FDR < 0.1); and (ii) very significantly up- (red) and downregulated (blue) metabolites (FC > 10 and FDR < 0.01). Note that very significantly up- and downregulated metabolites are subsets of up- and downregulated metabolites, respectively. Numbers shown in the upper-left and -right corners of each panel represent counts of up- and downregulated metabolites, respectively. Numbers in parentheses represent counts of very significantly up- or downregulated metabolites.

Volcano plots were generated to display differentially abundant metabolites using both loose (fold change (FC) > 2 and false discovery rate (FDR) < 0.1) and stringent (FC > 10 and FDR < 0.01) criteria. PSC, NC, and DC were compared in a pairwise fashion for both portal serum (Figure 2) and bile (Figure 3). There were considerable differences in metabolomic features between PSC and NC portal serum (112 metabolites were decreased and 39 metabolites were increased in abundance in PSC; Figure 2A). Notably, only two metabolites in portal serum were differentially abundant in PSC vs. DC (bilirubin and methylphosphate decreased and increased in PSC, respectively; Figure 2B). Substantial differences in metabolic features between DC and NC portal serum were also observed (157 metabolites were decreased and 81 metabolites were increased in abundance in DC; Figure 2C). Volcano plots of portal serum lipidomics revealed considerable differences between PSC and NC (49 lipid species decreased and 77 lipid species increased in abundance in PSC; Figure 2D). No significant differences were observed in lipidomics between PSC and DC patient portal serum (Figure 2E), and a total of 221 metabolites were differentially abundant (54 decreased and 167 increased) in DC portal serum compared to NC (Figure 2F).

Volcano plots were also generated to compare bile metabolomics and lipidomics in a pairwise fashion between the three patient cohorts. Considerable differences in metabolomic features were observed between PSC patient and NC bile (134 metabolites were decreased while 268 were increased in abundance in PSC; Figure 3A). Differences in the bile metabolome were also noted between PSC and DC (66 metabolites were decreased, while 107 metabolites were increased in abundance in PSC; Figure 3B). DC bile metabolome was also compared to NC; here, 118 metabolites were decreased while 227 were increased in abundance in DC (Figure 3C). Pairwise comparison of the bile lipidomic profiles also revealed substantial differences between PSC and NC (69 complex lipid species were decreased, while 805 were increased in PSC; Figure 3D). While no significant differences were detected in PSC vs. DC bile, mainly due to failure to pass the FDR threshold, four chemical species of the same lipid family (lactosylceramides (LCER)) exhibited >20-fold increased abundance in PSC vs. DC (mainly due to higher detection rate in PSC compared to DC bile; Figure 3E). Finally, the pairwise comparison between DC patient and NC bile revealed 54 decreased lipid species and 785 increased lipid species in DC (Figure 3F). Our entire (two-tailed) Mann–Whitney *U* test results for all annotated metabolites/lipids, for both portal serum and bile, are presented in Tables S1–S4.

**Figure 4:**
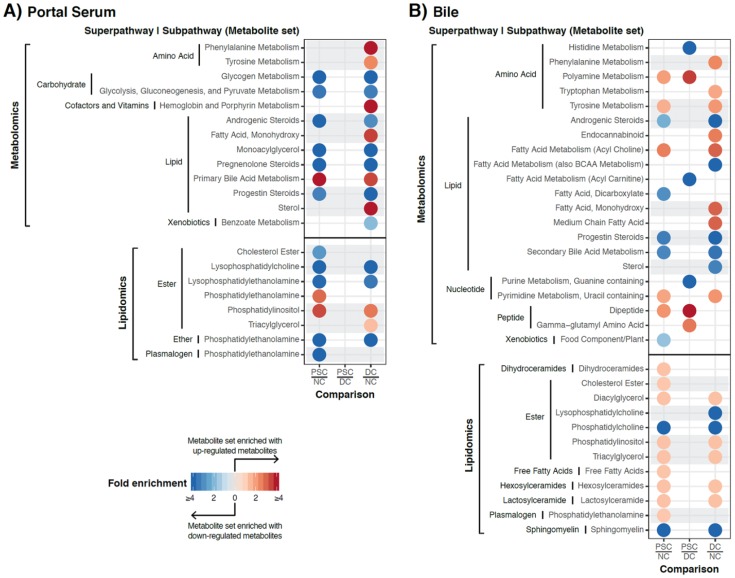
Overview of metabolite set enrichment analysis results for portal serum (**A**) and bile (**B**). Subpathways (i.e., metabolite sets) significantly enriched with up- or downregulated metabolites were identified using a hypergeometric test (*p* < 0.05) and shown with circles for the corresponding phenotype comparison. Color reflects the direction of the overall change (red for upregulation and blue for downregulation), while the intensity reflects the degree of enrichment compared to random chance. Subpathways enriched in both portal serum and bile (in at least one of the phenotype comparisons) were highlighted with gray background.

Having demonstrated that there is a large number of differentially abundant metabolites and complex lipids in portal serum and bile, we next performed metabolite set enrichment analysis (MSEA) to identify over-represented metabolite sets among the metabolites described above (based on the loose criteria). We identified 21 metabolite categories (sets) in patient portal serum that were enriched with differentially abundant metabolites or complex lipids in pairwise comparisons (Figure 4A). As expected from the volcano plot in Figure 2B, no categories were enriched between PSC and DC. However, nine categories characterized the metabolites or complex lipid species deficient in both PSC and DC portal serum compared to NC. These included: glycogen metabolism, glycolysis, gluconeogenesis, and pyruvate metabolism, androgenic, pregnenolone, and progestin steroids, monoacylglycerols, lysophosphatidylcholines, lysophosphatidylethanolamines, and phosphatidylethanolamines (ether form). In contrast, primary bile acids and phosphatidylinositols were enriched in portal serum in both PSC and DC compared to NC.

MSEA on bile samples also revealed that both PSC and DC patient bile had decreased abundance of secondary bile acids, androgenic and progestin steroids, phosphatidylcholines, and sphingomyelins compared to NC (Figure 4B). In contrast, both PSC and DC bile exhibited increases in chemical species of tyrosine metabolism, acyl choline fatty acid metabolism, uracil-containing pyrimidine metabolism, diacyl- and triacylglycerols, phosphatidylinositol, hexosyl- and lactosylceramides. Interestingly, PSC bile exhibited enrichment for polyamine metabolism and dipeptide metabolism as compared to both DC and NC (Figure 4B).

### 2.3. Identification of Differentially Abundant Metabolites or Lipids within Enriched Metabolite Sets

**Figure 5:**
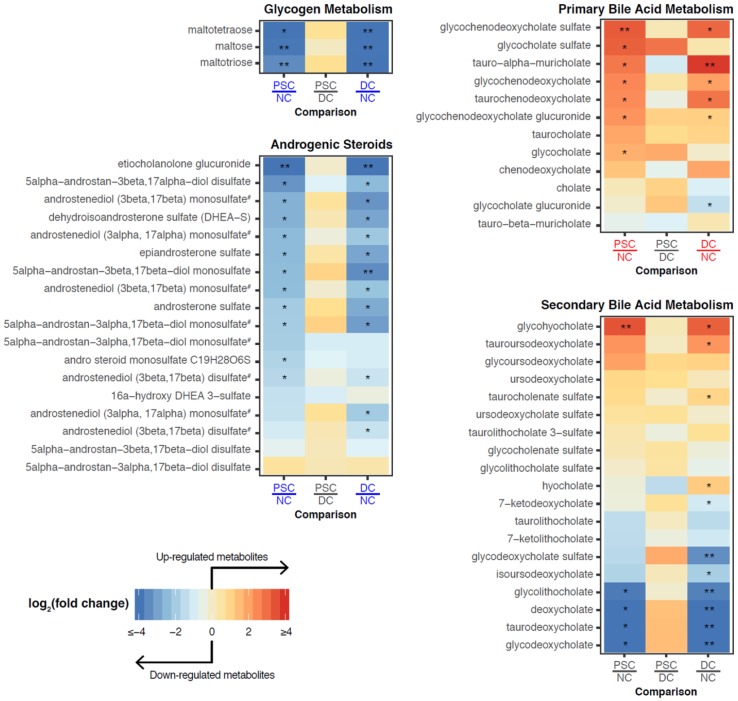
Heatmaps showing fold changes of individual metabolites from portal serum belonging to the subpathways of interest. Labels for phenotype comparisons were colored according to the metabolite set enrichment analysis results. Specifically, a label is colored red (blue) if the subpathway is significantly enriched with upregulated (downregulated) metabolites for the comparison. Metabolites that passed our criteria for differential abundance are marked with either a single asterisk (FC > 2 and FDR < 0.1) or double asterisks (FC > 10 and FDR < 0.01). ^#^ Metabolites measured more than once on the same LC-MS platform but with different retention time/index.

We next focused our analyses onto the specific metabolites comprising enriched metabolite sets in portal serum (Figure 5 and Figure S1). Glycogen breakdown products maltotetraose, maltotriose, and maltose were significantly lower in serum from both PSC and DC groups as compared to NC (Figure 5). Although this metabolite category was not found to be enriched in bile, the individual metabolites of this category trend towards being highly elevated exclusively in PSC patient bile (Figure S2).

Altered steroid/sterol metabolism was observed in PSC and DC patient portal serum as compared to NC (Androgenic Steroids shown in Figure 5); however, cholesterol levels were not altered in portal serum (data not shown). The levels of cholesterol-derived steroid hormones, including several sulfated hormones (e.g., dehydroepiandrosterone sulfate (DHEA-S; Figure 5) and pregnenolone sulfate (Figure S1)), were decreased in serum when comparing PSC and DC cohorts to NC.

Consistent with the clinical manifestation of liver diseases, the levels of primary bile acids were significantly elevated in portal serum from patients with liver disease (PSC and DC), particularly chenodeoxycholic acid derivatives (Figure 5). Similarly, significant elevations were noted in the conjugated secondary bile acid derivatives of the trihydroxy bile acid hyocholate (glycohyocholate) in patients with liver disease (Secondary Bile Acid Metabolism in Figure 5). Despite not passing our metabolite set enrichment criteria, several gut microbial metabolism-dependent secondary bile acids (e.g., deoxycholate, glycolithocholate) were decreased in PSC and DC compared to NC (Figure 5).

**Figure 6:**
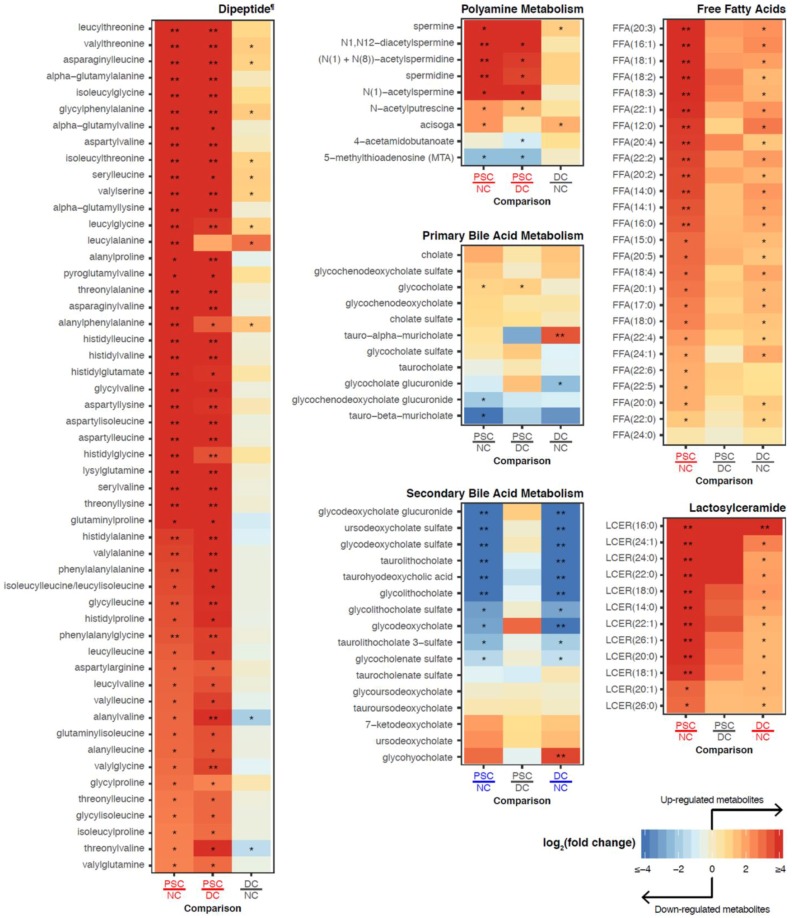
Heatmaps showing fold changes of individual metabolites from bile belonging to subpathways of interest. Labels for phenotype comparisons were colored according to the metabolite set enrichment analysis results. Specifically, a label is colored red (blue) if the subpathway is significantly enriched with upregulated (downregulated) metabolites for the comparison. Metabolites that passed our thresholds for differential abundance were marked with a single asterisk (FC > 2 and FDR < 0.1) or double asterisks (FC > 10 and FDR < 0.01). ^¶^ Due to the large size of this group, some dipeptides with smaller fold changes are not shown in this figure. For the full heatmap of all dipeptides, see Figure S2.

From our heatmaps of individual metabolites comprising metabolite sets of interest from bile (Figure 6 and Figure S2), we found that bile dipeptides were highly increased exclusively in PSC subjects (Figure 6 and Figure S2). In addition, leucine, isoleucine, and valine levels were uniquely elevated in PSC patient bile, while their downstream metabolites (e.g., isobutyrylcarnitine (C4), triglylcarnitine (C5:1-DC), isovalerylcarnitine (C5)) were decreased (Figure S2). Unique to PSC patients were also the highly elevated levels of polyamine metabolites in bile (e.g., spermidine, N1,N12-diacetylspermine, N-acetylputrescine, (N(1) + N(8))-acetylspermidine; Figure 6).

In contrast to the elevated primary bile acids in portal serum, the biliary primary bile acids were relatively similar across PSC, DC, and NC, with the exception of elevated tauro-alpha-muricholate in DC. Conversely, similar to portal serum, bile exhibited a decrease in secondary bile acids (e.g., taurolithocholate and glycolithocholate) in both DC and PSC compared to NC (Figure 6).

In both PSC and DC bile samples, profound changes were noted in lipid metabolism. Free fatty acids (FFAs) were increased in PSC and DC patients (e.g., FFA(20:3), FFA(16:1); Figure 6). Additionally, tri-acylgycerol levels were elevated in PSC and DC groups, while several classes of phospholipids were decreased (e.g., phosphatidylcholine; Table S1). Interestingly, lactosylceramide (LCER) levels were elevated in both PSC and DC bile compared to NC bile. Several of these LCER species were elevated in PSC samples compared to DC (e.g., LCER(16:0), LCER (24:1); Figure 6), despite the entire class not passing our FDR criteria in the PSC vs. DC pairwise comparison. In fact, none of these species was detected in bile from NC subjects, and only a subset of this class was detected in DC subjects.

### 2.4. Comparisons of Patient-Matched Serum and Bile Samples

**Figure 7:**
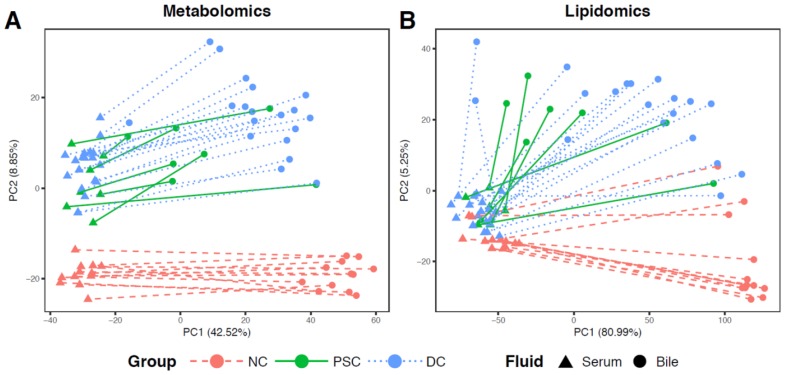
PCA plots of participant-matched portal serum and bile samples based on (**A**) metabolites and (**B**) complex lipids. Serum and bile samples from the same patient are linked with a red dashed line, green solid line, and blue dotted line for NC, PSC, and DC, respectively.

In an effort to directly assess global differences in enterohepatic circulation between disease groups, we performed PCA and mapped the participant-matched samples (serum and bile) onto ordination plots using features that were shared between both biological fluids (488 metabolites and 893 complex lipids; Figure 7). Similar to the previous PCA plots (Figure 1), NC portal serum samples (red triangles) clustered together and were apart from liver disease serum samples (PSC (green triangles) and DC (blue triangles)). Plotting of the matched NC bile samples (red circles) shows clear separation from NC portal serum along PC1. Interestingly, PSC patient serum samples and bile samples (green circles) generally separated far less from each other, compared to those of the other two phenotypes. Participant-matched PCA ordination plots of lipidomic data provided similar observations (Figure 7B), although separation between NC and diseased liver samples in serum was marginal.

## 3. Discussion

This study is the first to profile concurrent portal serum and biliary metabolomes in patients with end-stage PSC in order to evaluate alterations in and disruptions to enterohepatic circulation. The major findings include that: (i) portal serum metabolome was similar between PSC and DC patients, yet exhibited differential enrichment in several categories of metabolites (metabolite sets) compared to NC; (ii) PSC bile exhibited unique enrichment of dipeptides and metabolites of polyamine metabolism; and (iii) matched portal serum and biliary metabolomes of patients with PSC displayed greater homogeneity than either DC or NC. In addition to their potential implications for the pathophysiology of PSC, many of the identified metabolites may potentiate downstream effects on the fecal microbiome, either as substrates or as inhibitors of microbial activity. Together, these findings inform on metabolic pathways that may contribute to an altered metabolomic footprint in enterohepatic circulation during late-stage liver disease. Moreover, these results provide the impetus for novel lines of investigation towards the development of novel experimental therapies for PSC.

PSC and DC patients exhibited differences in carbohydrate metabolism compared to NC, as exemplified by the decrease in portal serum glycogen breakdown products (i.e., maltose, maltotriose, and maltotetraose), with a trend towards a unique increase of these products in PSC bile. Cirrhosis has previously been demonstrated to lead to increased gluconeogenesis and decreased glycogenolysis in the liver [22,23]. Our results further suggest that cirrhotic patients exhibit either decreased amylase hydrolysis of dietary starch in the gut or diminished absorption of these glucose polymers into portal circulation. Cholesterol-derived steroids were also decreased in the portal serum of both PSC and DC patients compared to NC, including DHEA-S and 3-hydroxy-3-methylglutarate (HMG; Table S1). Interestingly, both DHEA-S and HMG may have roles in fibrosis; lower DHEA-S levels positively correlate with fibrosis in nonalcoholic fatty liver disease and idiopathic pulmonary fibrosis [24,25]. HMG-CoA reductase inhibitors, which inhibit HMG conversion to cholesterol, have been associated with slower progression of liver fibrosis [26,27]. While it is unclear precisely how HMG-CoA reductase inhibitors alter fibrosis pathways, they have both direct and indirect pleitropic effects [28].

End-stage PSC and DC patients both demonstrate enrichment in conjugated forms of chenodeoxycholic acid in their portal serum. This is consistent with the reduction in microsomal sterol 12α-hydroxylase (CYP8B1) levels that have previously been observed in the setting of PSC cirrhosis [29]. Conversely, some secondary bile acids are decreased in both PSC and DC cohorts, suggesting a reduction in the microbial population capable of bile salt hydrolase and other secondary bile acid enzymatic activities. The increase in tauro-muricholic acid also reflects a shift in microbial ecology, as it is normally metabolized by the fecal microbiome and is incidentally an antagonist of the farnesoid X receptor (FXR) [30]. Additionally, the free fatty acid metabolite set was solely enriched in the bile of patients with PSC compared to NC. Studies in nonalcoholic liver disease demonstrate that FFAs can disrupt short heterodimer partner (SHP)-induced FXR activation, leading to the upregulation of NTCP and CYP7A1, thereby permitting unregulated bile acid synthesis and subsequent hepatic injury [31]. In PSC, there is a lack of CYP7A1 suppression despite high levels of SHP and FXR gene expression, supporting the presence of dysregulated SHP signaling [29]; however, this may not be a generalizable feature of PSC, as very low levels of serum C4 (an indicator of CYP7A1 enzymatic activity) were detected in the advanced stages of PSC [32]. The elevated levels of FFAs may indicate possible defects in β-oxidation, which is also enabled by FXR as well as peroxisome proliferator-activated receptor-alpha (PPAR-α) [33,34]. Of interest, we did not observe an elevation of bilirubin in the portal serum of PSC patients, which, while purely speculative, may also reflect an alteration in gut microbiota composition, as demonstrated in a rodent model [35].

Based on our data, the lipidomic profiles in both portal serum and bile were similar between end-stage DC and PSC patients. While somewhat surprising, this observation may reflect that all liver disease patients had end-stage liver disease. As cirrhosis is the final common pathway of all liver disease, it may be that initial alterations in both portal blood and bile eventually converge (reflecting the extreme hepatic dysfunction); hence leading to lipidomic similarities among end-stage patients. It should be noted, however, that the bioactive sphingolipid, LCER, was significantly increased in PSC and DC bile as compared to NC, with a trend towards more substantial increases, exclusively in PSC. LCER is not only a key substrate in glycosphingolipid synthesis, but it also plays an integral role in cellular homeostasis [36]. LCER is a lipid second messenger that can induce a proinflammatory state through upregulation of inducible nitric oxide synthase (iNOS), PI3kinase/Akt, and superoxides [36]. Induction of nitric oxide can lead to cholestasis and damage to the biliary epithelium by impairing cAMP-dependent ion transport [37,38,39], while activation of PI3kinase/Akt can lead to biliary proliferation and activation of hepatic stellate cells [40,41]. LCER may also induce superoxide production in neutrophils [42], and may even disrupt lysosomal function, with levels increased in lysosomal storage diseases such as Niemann–Pick [43]. Whether LCER is involved in the initiation or progression of PSC remains to be investigated, yet the effects of LCER on cellular processes and pathways are amenable to laboratory investigation.

PSC bile uniquely exhibited elevated levels of dipeptides. Normally, peptides and amino acids comprise approximately 5% of bile, with glutathione and glutamic acid being the major contributors [44]. Transport of peptides occurs via organic anion transport polypeptide (OATP), but can also be facilitated by conjugation with bile acid moieties [45,46,47,48]. In silico work has demonstrated that the affinity between peptides and bile acids is variable, with tyrosine-, tryptophan-, phenylalanine-, leucine-, isoleucine-, and valine-containing peptides having a twofold greater binding activity for bile acids [49]. While the mechanism behind the high biliary excretion of dipeptides in PSC patient bile is unclear, it is notable that nearly 75% of the enriched dipeptides are composed of at least one of these high-affinity amino acids. A potential source of biliary dipeptides is increased protein catabolism, yet patients with moderately advanced PSC have lower peripheral serum levels of dipeptides than healthy controls, arguing against elevated catabolism [20]. Further research is needed to determine the mechanism of higher hepatic excretion of dipeptides into bile, whether this reveals insight into hepatocellular dysfunction in PSC, and if this might be the mechanism behind low serum dipeptide levels in PSC.

Similar to dipeptides, polyamines, including acetylated forms, were elevated exclusively in PSC bile, with reductions only in 5-methylthioadenosine (MTA), a metabolite involved in polyamine biosynthesis. Polyamines are a group of ubiquitous polycations that interact with negatively charged molecules, including RNA, DNA, and phospholipids. This group of molecules play a variety of roles in cellular homeostasis, including protein synthesis, and cell growth, survival, and proliferation [50,51]. While these molecules are essential for normal cellular function, it is now recognized that catabolism of polyamines is associated with the cellular efflux of these molecules [52] and the production of reactive oxygen species (ROS), including hydrogen peroxide and aldehydes [53]. Indeed, polyamine catabolism and the production of ROS has been implicated in disease progression (e.g., ischemic reperfusion injury [53]). Moreover, MTA is a byproduct of S-adenosylmethionine (SAMe) metabolism and, like SAMe, it can act as a primary methyl donor for DNA and protein methylation [54,55]. MTA also has anti-inflammatory and antiproliferative effects [54,55]. Whether polyamine catabolism is involved in the pathophysiology of PSC remains to be explored, but is testable in in vitro and in vivo models of disease.

In addition to their direct effects on the hepatic microenvironment, many of the observed altered metabolites, including polyamines (and MTA), dipeptides, and FFA, can alter microbial function and thus may have global effects on enterohepatic circulation. While polyamine catabolism in mammalian cells can generate free radicals and aldehyde, polyamines are important mediators against oxidative stress in prokaryotes, and may permit scavenging of nucleotides and reactive oxygen species, as well as expression of enzymes such as superoxide dismutase [56]. Exogenous sources of polyamines can be utilized by bacteria, and can induce negative feedback on the microbial catabolism of SAMe and MTA [56]. Both SAMe and MTA are integral to the bacterial production of methionine and organic sulfur [57]. Interestingly, dipeptides can be metabolized by numerous bacteria, with variable effects on bacterial growth depending on the presence of specific dipeptidases. In a setting of minimal peptidase activity, high levels of dipeptides can be bacteriostatic or even bactericidal [58]. Additionally, certain bacteria can incorporate exogenous FFA into their membranes, and *Enterococcus faecalis* has even been shown to alter its membrane composition based on the availability of specific FFAs, which can, in turn, modify its ability to survive stressors such as higher bile flow or antibiotic exposure [59,60]. Hence, altered bile content may modify the human gut environment, leading to changes in bacterial communities, microbiota functional output, and, potentially, gut homeostasis.

Lastly, our study demonstrates that, in patients with PSC, portal serum and bile from the same individuals are relatively homogeneous as compared to matched samples from DC or NC. This may highlight defects in cannalicular secretion, or even alterations in cholehepatic shunting due to shifts in bile acid content. Cholehepatic shunting is hypothesized to increase choleresis via bile salt-dependent and -independent means, and enhancing bile flow and bicarbonate secretion into the biliary tree [61]. Interestingly, the apical sodium bile salt transporter (ASBT), which is responsible for cholehepatic shunting, has higher affinity for conjugated bile acids, particularly dihydroxy bile acids, such as chenodeoxycholic acid (which was higher in PSC and DC portal serum), rather than trihydroxy bile acids, such as derivatives of cholic acid [62].

Due to the small sample sizes of our study groups, our findings require further validation. For example, while PSC and DC patients met end-stage liver disease criteria (e.g., biliary cirrhosis in PSC patients), DC patients had significantly higher MELD scores, which may be a confounding factor. Additionally, we chose to obtain bile from the gallbladder rather than the bile duct, since doing so is less invasive and minimizes study-related risk. We recognize that gallbladder abnormalities are common in PSC patients, and the metabolomic profile of PSC patient gallbladder bile may be confounded by gallbladder inflammation and stasis [63]. It should be noted, however, that abnormal gallbladder inflammation may also reflect part of the disease spectrum in PSC, as up to 25% of PSC patients may have cholecystitis [64]. Moreover, given the small sample size, we did not stratify the groups based on sex or age, both of which may contribute to differences in metabolomic profiles. Likewise, we did not separate and compare portal serum and bile from PSC patients with and without concurrent IBD. This comparison, including IBD-only patient samples, would enable us to detect altered metabolomic profiles specific to PSC, and not the result of IBD. Future studies should carefully consider these possible confounders with sufficient sample size. However, expansion of sample size in this unique patient cohort is slowed by being limited to collections during living donor transplants and the challenges of obtaining portal blood. Moreover, if possible, further studies of portal serum and bile metabolomes from patients with early- to moderate-stage PSC might reveal even more striking changes. Notably, it is not possible to determine whether the changes demonstrated in the metabolome are a consequence of PSC, or are causative of PSC. Thus, future experimental approaches would be dependent on alternative models such as murine, cell cultures, and organoids. Furthermore, future studies would benefit from multiomic approaches using peripheral blood, fecal, and urine metabolomics, as well as stool metagenomics. In conclusion, our results demonstrate that the biliary metabolome of patients with PSC is significantly altered. Many of these changes are shared with patients with noncholestatic cirrhosis, suggesting that end-stage liver disease may cause a convergence of metabolic derangements. Even so, PSC bile exhibits significant increases in dipeptide and polyamine metabolites, which can alter hepatic homeostasis as well as the intestinal microbiota. These observations provide insights into additional hypothesis-testing experiments and potential therapeutic pathways.

## 4. Materials and Methods

### 4.1. Study Design and Population

This study was approved by the Mayo Clinic Institutional Review Board (IRB #13-001312) following the rules of the 1975 Declaration of Helsinki. Adult participants who provided consent were prospectively enrolled from the Mayo Clinic Liver Transplant inpatient and outpatient services between August 2013 and December 2014. Participants were eligible if they were scheduled to undergo a living donor liver transplant for PSC or other chronic liver disease, or were live donors for liver transplant. Patients were excluded if they met the following criteria: (i) history of concomitant liver disease (i.e., PSC with chronic viral hepatitis), (ii) prior organ transplant, (iii) renal failure requiring hemodialysis. In addition, patients were excluded if they were treated with an investigational drug, or had an acute gastrointestinal illness (i.e., infectious colitis, flare of IBD) in the previous 6 months. Charts were reviewed to confirm patient eligibility. Portal serum and bile was collected from: (i) 7 patients with PSC, (ii) 19 patients with non-PSC cirrhosis (DC), and (iii) 12 donors (NC)

### 4.2. Sample Accessioning and Preparation

Portal blood (4 mL) and gallbladder bile (2 mL) were collected intraoperatively. Bile was immediately placed on ice, promptly divided into 100 μL aliquots, and frozen at −80 °C. Blood was similarly placed on ice, promptly fractionated by centrifugation, divided into 100 μL aliquots, and stored at −80 °C. One-hundred microliters serum and bile aliquots from each participant were sent to Metabolon Inc. for further processing. Briefly, the samples were prepared using the MicroLab STAR^®^ system (Hamilton Company, Reno, NV, USA). Metabolite extraction was optimized (i.e., removal of protein, dissociation of small molecules bound to protein or trapped in the precipitated protein matrix, recovery of chemically diverse metabolites) by precipitating protein with methanol using vigorous agitation for 2 min (Glen Mills GenoGrinder 2000, Clifton, NJ, USA) followed by centrifugation. The resulting extract was divided into 5 fractions: (i) 2 for analysis by 2 separate reverse phase (RP)/UPLC-MS/MS methods with positive-ion mode electrospray ionization (ESI), (ii) 1 for analysis by RP/UPLC-MS/MS with negative-ion mode ESI, (iii) 1 for analysis by HILIC/UPLC-MS/MS with negative-ion mode ESI, and (iv) 1 sample reserved for backup. Organic solvent was removed using a TurboVap^®^ (Zymark Corporation, Massachusetts, USA). Sample extracts were stored overnight under nitrogen before preparation for analysis.

### 4.3. Sample Analysis

#### 4.3.1. UPLC-MS/MS

All methods described below utilized Waters ACQUITY UPLC and a Thermo Scientific Q-Exactive high resolution/accurate mass spectrometer interfaced with a heated electrospray ionization (HESI-II) source and Orbitrap mass analyzer operated at 35,000 mass resolution. The MS analysis alternated between MS and data-dependent MS^n^ scans using dynamic exclusion. The scan range varied slightly between methods but covered 70–1000 *m*/*z*. The sample extract was dried, and then reconstituted in solvents compatible to each of the four methods. Each reconstitution solvent contained a series of standards at fixed concentrations to ensure injection and chromatographic consistency. Analysis of each of the four aliquots was performed to optimize capture of specific metabolites, as follows: (i) Acidic positive ion conditions (chromatographically optimized for hydrophilic compounds): The extract was gradient eluted from a C18 column (Waters UPLC BEH C18-2.1 × 100 mm, 1.7 µm) using water and methanol, containing 0.05% perfluoropentanoic acid (PFPA) and 0.1% formic acid (FA), (ii) Acidic positive ion conditions (chromatographically optimized for hydrophobic compounds): The extract was gradient eluted from the same aforementioned C18 column using methanol, acetonitrile, water, 0.05% PFPA and 0.01% FA and was operated at an overall higher organic content, (iii). Basic negative ion conditions: The extracts were gradient eluted from a separate, dedicated C18 column using methanol and water with 6.5 mM Ammonium Bicarbonate (pH 8), (iv) Negative ionization: The extracts were gradient eluted from a HILIC column (Waters UPLC BEH Amide 2.1 × 150 mm, 1.7 µm) using a gradient consisting of water and acetonitrile with 10mM Ammonium Formate (pH 10.8).

#### 4.3.2. Complex Lipids Platform

Lipids were extracted from samples in methanol:dichloromethane in the presence of internal standards. The extracts were concentrated under nitrogen and reconstituted in 0.25 mL of 10 mM ammonium acetate dichloromethane:methanol (50:50). The extracts were transferred to inserts and placed in vials for infusion-MS analysis, performed on a Shimazdu LC with nano-PEEK tubing and the Sciex SelexIon-5500 QTRAP. The samples were analyzed via both positive- and negative-mode electrospray. The 5500 QTRAP scan was performed in MRM mode with a total of more than 1100 MRMs. Individual lipid species were quantified by taking the peak area ratios of target compounds and their assigned internal standards, then multiplying by the concentration of internal standard added to the sample. Lipid class concentrations were calculated from the sum of all molecular species within a class, and fatty acid compositions were determined by calculating the proportion of each class comprised by individual fatty acids.

### 4.4. Quality Control

Experimental samples were analyzed together with 3 different control samples: (i) technical replicates consisting of a sample pooled from each experimental sample (or alternatively, well-characterized human plasma), (ii) process blanks, consisting of extracted water, (iii) a sample to monitor instrument performance and chromatographic alignment, consisting of a cocktail of QC standards that were carefully chosen not to interfere with the measurement of endogenous compounds (spiked into every sample). Instrument variability was determined by calculating the median relative standard deviation (RSD) for the standards that were added to each sample prior to injection into the mass spectrometers. Overall process variability was determined by calculating the median RSD for all endogenous metabolites (i.e., noninstrument standards) present in 100% of the pooled matrix samples.

### 4.5. Data Extraction and Compound Identification

Raw data were extracted, peak-identified, and QC-processed using proprietary hardware and software (Metabolon, Inc.). Compounds were identified using library entries of purified standards or recurrent unknown entities. Metabolon maintains a library based on authenticated standards that contains, for all molecules present in the library, the retention time/index (RI), mass to charge ratio (*m/z*), and chromatographic data (including MS/MS spectral data). Furthermore, biochemical identifications were based on 3 criteria: retention index within a narrow RI window of the proposed identification, accurate mass match to the library +/−10 ppm, and the MS/MS forward and reverse scores between experimental data and authentic standards. The MS/MS scores were based on a comparison of the ions present in the experimental spectrum to the ions present in the library spectrum. While there may have been similarities between these molecules based on one of these factors, the use of all 3 data points could be utilized to distinguish and differentiate biochemicals. More than 3300 commercially available purified standard compounds were acquired and registered for analysis on all platforms for determination of their analytical characteristics.

### 4.6. Statistical Calculations

Statistical analyses were performed on raw metabolomic and lipidomic data provided by Metabolon, Inc. For each sample, original raw peak (or area) intensities were normalized into relative intensities in order to minimize the potential effect of confounding factors (i.e., variance in total metabolite concentrations across samples) and to enable comparisons across serum and bile. Normalization was performed by dividing the original data into 5 matrices corresponding to the 5 different LC-MS runs (i.e., 4 UPLC-MS/MS columns for metabolomics and 1 DMS-MS column for lipidomics). Missing values were replaced with sample minimum values in each matrix; intensity values within were then divided by the sum of all values of that particular sample within each matrix (i.e., total intensity normalization). Then, the 4 matrices for UPLC-MS/MS metabolomic data were merged to yield a combined metabolomic data matrix, while the 1 remaining DMS-MS matrix served as a lipidomic data matrix. Finally, log_2_ transformation was applied to both matrices to yield the final data matrices (Tables S5–S8). Notably, this samplewise normalization procedure equally weighted each LC-MS run. This procedure was applied separately for portal serum and bile samples.

A two-tailed Mann–Whitney *U* test was applied to identify differentially abundant metabolites. *p*-values from the test were subjected to the Benjamini–Hochberg procedure for FDR control. To identify differentially abundant metabolites, we used two different criteria, which were based on different thresholds for FC and FDR: loose (FC > 2 and FDR < 0.1) and stringent (FC > 10 and FDR < 0.01) criteria. MSEA was conducted to identify over-represented metabolite categories for the differentially abundant metabolites based upon our loose criteria. Metabolite categories were defined according to the subpathway annotations provided by Metabolon; over-representation analysis was performed using a hypergeometric test implemented in R [65]. A subpathway (metabolite category) with a *p*-value < 0.05 was considered statistically significant.

## Figures and Tables

**Table 1:** Clinical characteristics of study participants.

	Primary Sclerosing Cholangitis	Non-Diseased Controls	Disease Controls
	(PSC, *n* = 7)	NC, *n* = 12)	(DC, *n* = 19)
**Demographics**			
Age (years), median (range)	37 (20–61)	34 (22–54)	58 (22–71)
Female (%)	43	25	16
BMI, median (range)	24 (20–26)	26 (22–30)	28 (20–43)
Diabetes (%)	0	0	21
IBD (%)	57	0	0
**Liver Biochemistries**			
ALT (U/L), median (range)	60 (20–742)	21 (13–68)	42 (22–745)
AST (U/L), median (range)	70 (15–884)	21 (15–157)	62 (34–1730)
Alk Phos (U/L), median (range)	329 (186–1626) *^,#^	65 (45–95)	118 (34–255) *
Bilirubin total (mmol/L), median (range)	1.40 (0.4–12.2) *	0.7 (0.2–1.8)	3.5 (0.4–38.7) *
**Other Parameters**			
Leukocyte count (×10^3^/mL), median (range)	6.7 (1.9–9.0)	5.7 (3.5–9.0)	3.6 (1.7–7.9)
Colectomy (%)	0	0	0
ABO blood type	71% (O), 29% (AB)	91% (O), 9% (A)	32% (O), 53% (A), 10% (B)
MELD Score, median (range)	25 (13–31) ^#^	N/A	31 (19–40)
**Medications at TX**			
UDCA (%)	29	0	10
Antibiotics (%)	14	0	21

*, *p* < 0.05 vs. NC; ^#^, *p* < 0.05 vs. DC. Corrected, as needed, for the number of comparisons made (Bonferroni-Holm correction).

## Supplementary Materials

Supplementary materials are available online at http://www.mdpi.com/1422-0067/19/10/3188/s1. Figure S1. Heatmaps of additional portal serum metabolite sets. ^#^ Metabolites measured more than once on the same LC-MS platform but with different retention time/index. Figure S2. Heatmaps of additional bile metabolite sets. Table S1. Mann–Whitney (two-tailed) *U* test results for identification of differentially abundant metabolites in portal serum metabolomic data. Table S2. Mann–Whitney (two-tailed) *U* test results for identification of differentially abundant complex lipids in portal serum lipidomic data. Table S3. Mann–Whitney (two-tailed) *U* test results for identification of differentially abundant metabolites in bile metabolomic data. Table S4. Mann–Whitney (two-tailed) *U* test results for identification of differentially abundant complex lipids in bile lipidomic data. Table S5. log_2_-transformed relative intensities (total intensity normalization) for chemicals in portal serum metabolomic data. Table S6. log_2_-transformed relative intensities (total intensity normalization) for chemicals in portal serum lipidomic data. Table S7. log_2_-transformed relative intensities (total intensity normalization) for chemicals in bile metabolomic data. Table S8. log_2_-transformed relative intensities (total intensity normalization) for chemicals in bile lipidomic data.
