# Supplementary figures and images for: Metabolomic Profiling of Portal Blood and Bile Reveals Metabolic Signatures of Primary Sclerosing Cholangitis
Source: Int J Mol Sci. 2018 Oct 16;19(10):3188. doi: 10.3390/ijms19103188 (PMC6214107; doi:10.3390/ijms19103188)

# Figure S1

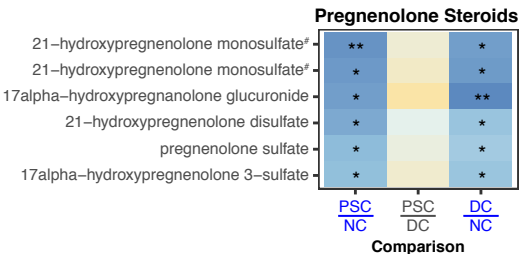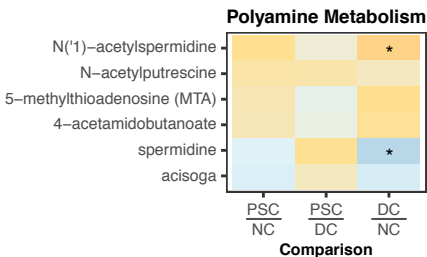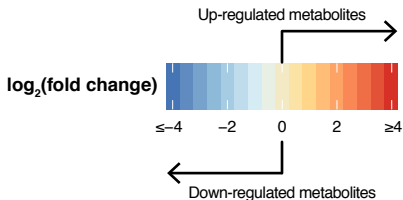

# Figure S2

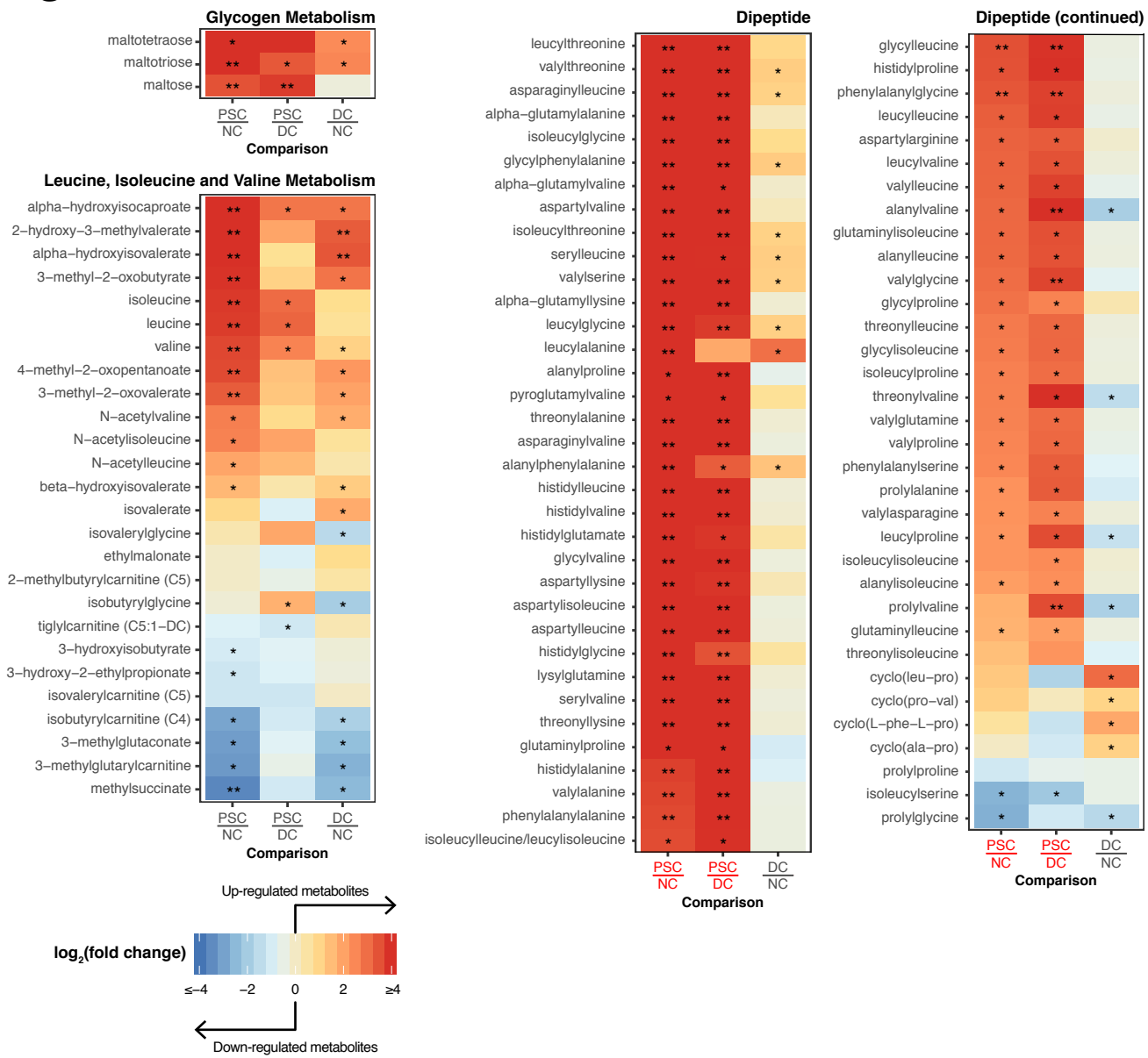

Supplement: Supplementary file 1 [file ijms-19-03188-s001.zip › Suppl Figs 1-2 Labelled.pdf]
